# Comparative analysis of four commercial on-farm culture methods to identify bacteria associated with clinical mastitis in dairy cattle

**DOI:** 10.1371/journal.pone.0194211

**Published:** 2018-03-15

**Authors:** Jair C. Ferreira, Marilia S. Gomes, Erika C. R. Bonsaglia, Igor F. Canisso, Edgar F. Garrett, Jamie L. Stewart, Ziyao Zhou, Fabio S. Lima

**Affiliations:** Department of Veterinary Clinical Medicine, College of Veterinary Medicine, University of Illinois, Champaign-Urbana, IL, United States of America; Universita degli Studi di Napoli Federico II, ITALY

## Abstract

Several multiple-media culture systems have become commercially available for on-farm identification of mastitis-associated pathogens. However, the accuracy of these systems has not been thoroughly and independently validated against microbiological evaluations performed by referral laboratories. Therefore, the purpose of the present study was to evaluate the performance of commercially available culture plates (Accumast, Minnesota Easy System, SSGN and SSGNC Quad plates) to identify pathogens associated with clinical mastitis in dairy cows. Milk samples from the affected quarter with clinical mastitis were aerobically cultured with the on-farm culture systems and by two additional reference laboratories. Agreeing results from both standard laboratories were denoted as the reference standard (RS). Accuracy (Ac), sensitivity (Se), specificity (Sp), positive and negative predictive values (PPV and NPV, respectively) and Cohen’s kappa coefficient (*k*) of on-farm plates were determined based on the RS culture of 211 milk samples. All four plate-systems correctly identified ≥ 84.9% of milk samples with no bacterial growth. Accumast had greater values for all overall predictive factors (Ac, Se, Sp, PPV and NPV) and a substantial agreement (*k* = 0.79) with RS. The inter-rater agreements of Minnesota, SSGN, and SSGNC with RS were moderate (0.45 ≤ *k* ≤ 0.55). The effectiveness to categorize bacterial colonies at the genus and species was numerically different amongst the commercial plates. Our findings suggest that Accumast was the most accurate on-farm culture system for identification of mastitis-associated pathogens of the four systems included in the analysis.

## Introduction

After decades of advancements in the development of strategic prevention programs, mastitis remains one of the most prevalent and economically detrimental diseases in dairy farms worldwide [1, 2]. The inflammation of the mammary gland is a complex and multifactorial disorder [3] caused predominantly by coagulase-negative staphylococci, *Bacillus* spp., *Streptococcus* spp., *Staphylococcus aureus (S*. *aureus)*, and *Escherichia coli (E*. *coli)* [4]. Despite the growing concern regarding the use of antimicrobials in farm animals [5], mastitis remains the leading reason for antibiotic usage in dairy cows [6].

Even though the precise identification of mastitis agents is critical for determining therapeutic intervention [7], the laboratory investigations have not been routinely adopted due to their cost and the extended turnaround time for receiving culture results. On the other hand, on-farm selective media tests that identify Gram-positive and Gram-negative pathogens were developed in the last decades providing a less costly and faster alternative of laboratory culturing [8–10]. The principle of on-farm culture is rapid results that allow producers to make strategic treatment decisions for clinical cases at the farm. These tests have become an upcoming diagnostic tool and decision criterion for identification and treatment of clinical mastitis cases in dairy cows [8–10]. However, the antibiotic resistance [11, 12] and the probability of cure may vary among etiological agents belonging to the same bacteria group [13]. A body of evidence suggests that intramammary antibiotic therapy improves the rate of cure in cows infected with *Klebsiella* spp. [14], *Staphylococcus* spp. [15], and environmental streptococci [11, 12]. Whereas, the use of an intramammary antibiotic is not recommended for cows with mastitis associated with *E*. *coli* [16] and *S*. *aureus* [17, 18], except in cases of severe mastitis. Thus, on-farm identification of specific mastitis-related pathogens might be an important strategy to improve judicious use of antibiotics in dairy farms.

The development of a precise cow-side system for the diagnosis of mastitis pathogens is essential for quick therapeutic intervention. In the last decade, several multiple-media culture systems became available for on-farm identification of general levels of mastitis-related pathogens [8, 19, 20]. Besides the conventional differentiation among Gram-positive or Gram-negative pathogens, some on-farm plate systems also proposed a more specific diagnosis. The Minnesota Easy Culture System Tri-Plate (Minnesota), which contains a selective catalase-negative media, showed reasonable confidence to identify infections caused by staphylococci and streptococci [19]. Accumast system consists of three selective chromogenic media, and it was recently successful for diagnosis of specific mastitis-related agents, such as *Enterococcus* spp. and *S*. *aureus* [20]. Using Quad-plate tests, experienced readers correctly identified specific mastitis etiological agents with 86% of agreement to a standard laboratory method [21]. However, studies comparing the relative efficacy of distinct on-farm tests for microbiological profile characterizing of milk samples are currently limited [22]. Therefore, the purpose of the present study was to compare the performance of four commercial culture plate systems for the identification of specific clinical mastitis-pathogens in dairy cows and their categories (Gram-positive, Gram-negative, No growth).

## Materials and methods

### Ethics statement

The research protocol was reviewed and approved by the Institutional Animal Care and Use Committee of the University of Illinois (Protocol number: 15060).

Sampling animals that present abnormal milk during forestripping on milking preparation is also routine procedure at the study site.

### Milk sample collection

A cross-sectional study was performed to assess the efficacy of on-farm culture plate tests for identification of pathogens associated with clinical mastitis using a total of 299 milk samples from two commercial dairy herds located in central Illinois (n = 177) and New York State (n = 122) within a two-month interval between October and November of 2015. These herds were chosen because of relationship with principal investigator. Milk samples were aseptically collected by trained research personnel from mammary quarters with signs of clinical mastitis according to the guidelines of the National Mastitis Council [23]. Briefly, teats were cleaned and disinfected using 70% ethanol, the first three streams were discarded, and then milk samples were collected in sterile 50 mL conical tubes (Corning Life Sciences, Tewksbury, MA), homogenized, and distributed in three aliquots of approximately 10 mL of milk into sterile plastic tubes without preservative (Corning Life Sciences, Tewksbury, MA). A case of clinical mastitis was defined as the presence of abnormal milk (i.e., watery appearance, presence of flakes or clots) with or without cardinal signs of mammary gland inflammation (i.e., udder swelling, redness, painful, and heat upon udder palpation), both detected at each milking by the trained research personnel. Lactating cows that were determined to have severe clinical mastitis (i.e. rectal temperature ≥ 39.5°C, anorexia, marked depression) were not eligible for the study.

The first aliquot of each milk sample obtained in the herd located in the central Illinois and New York was placed in ice and transported to the respective laboratory at the University of Illinois and Cornell University and then plated within 2 hours after the sample was collected. The second and third aliquots were transported in ice and frozen at—20°C immediately at the arrival at the laboratory. The frozen aliquots were sent to the Quality Milk Production Services (QMPS; Cornell University, Ithaca, NY) and the Veterinary Diagnostic Laboratory (VDL; University of Illinois, Urbana, IL) for standard microbiological analysis.

### Plate diagnostic tests

All plate diagnostic tests were performed by trained personnel from the University of Illinois at the laboratory at University of Illinois for samples collected at Illinois herd or at Cornell University for samples collected at New York herd. Milk samples were cultured using four on-farm plate systems, which constitute the most popular commercially available options in the USA at the time when the study was performed: Accumast (FERA Animal Health LCC, Ithaca, NY), Minnesota Easy System Tri-Plate (University of Minnesota Laboratory for Udder Health, St. Paul, MN), Mastitis SSGN Quad plate (DQCI Services, Mounds View, MN) and Mastitis SSGNC Quad plate (DQCI Services, Mounds View, MN) by the trained laboratory personnel. Initially, a cotton swab was immersed into the sterile plastic tube containing the milk sample, and then wiped across in a zig-zag pattern onto the surface of each section of the selective growth media of the four plate systems in accordance to each manufacturer’s guidelines. A sterile cotton swab was used for each plate section. Plates were aerobically incubated at 37°C for 24 hours and subsequently read on-site by a single member of the research team. The presence of one colony of a specific pathogen was the criterion to classify the sample as positive for those specific bacteria.

**Fig 1:**
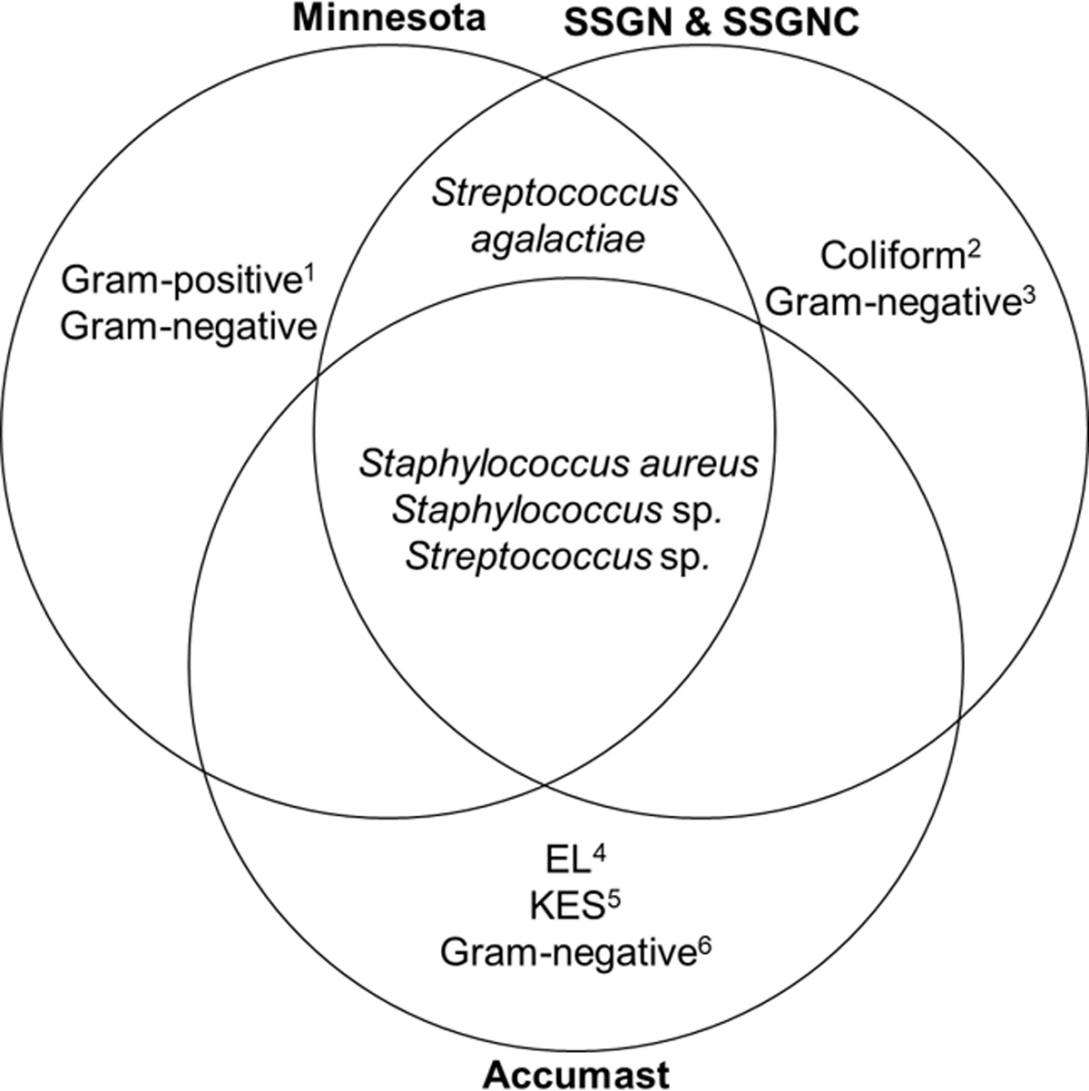
Venn diagram illustrating common mastitis-related pathogens amongst all plates (Minnesota Easy System Tri-Plate, Accumast, Mastitis SSGN Quad plate and Mastitis SSGNC Quad plates), or common mastitis-related pathogens present in at least two plates. ^1^ Except *S*. *aureus*, *Staphylococcus* spp., *Streptococcus agalactiae* and *Streptococcus* spp. ^2^ *Citrobacter* spp., *Klebsiella* spp., *Enterobacter* spp., and/or *Serratia* spp. ^3^ Except *E*. *coli* and coliforms. ^4^ *Enterococcus* spp. and/or *Lactococcus* spp. ^5^ *Klebsiella* spp., *Enterobacter* spp., and/or *Serratia* spp. ^6^ Except *E*. *coli* and bacteria from KES group.

The diagnosis of mastitis-related bacteria or group of bacteria was carried out according to the manufacturers' recommendations for the respective systems (Table 1 and Fig 1). The Minnesota Easy System Tri-plate utilizes three distinct types of culture media (Factor, MacConkey and modified TKT agar) to diagnose milk samples with no bacteria growth, Gram-negative bacteria, *S*. *aureus*, *Staphylococcus* spp., *Streptococcus agalactiae*, *Streptococcus* spp., and undefined Gram-positive bacteria. The Accumast system uses three selective chromogenic media to identify specific bacteria or group of bacteria. Accumast identifies *S*. *aureus*, *Staphylococcus* spp., *Streptococcus* spp., *Enterococcus* spp. or *Lactococcus* spp. (EL group), *Klebsiella* spp., *Enterobacter* spp. or *Serratia* spp. (KES group), *E*. *coli*, Gram-negative bacteria other than *E*. *coli* or KES, and milk samples with no bacterial growth. Both quad plates (SSGN and SSGNC) contained the same differential and selective bacterial growth media, except that an enzyme substrate specific for *E*. *coli* has been added to SSGNC. The levels of diagnosis of SSGN and SSGNC were *S*. *aureus*, *Staphylococcus* spp., *Streptococcus agalactiae*, *Streptococcus* spp., *E*. *coli*, coliforms others than *E*. *coli*, Gram-negative bacteria others than coliforms, and milk samples with no bacteria growth. Strains from the genera *Citrobacter*, *Enterobacter*, *Escherichia*, *Klebsiella*, and *Serratia* were considered as coliforms [24]. All plates were cultured for additional 24 hours and re-read before final results were recorded.

**Table 1:** Mastitis-associated pathogens of dairy cows identified by four commercial on-farm plate systems (Minnesota Easy System Tri-Plate, Accumast, Mastitis SSGN Quad plate and Mastitis SSGNC Quad plate).

Minnesota	Accumast	SSGN	SSGNC
*Staphylococcus* spp.	*Staphylococcus* spp.	*Staphylococcus* spp.	*Staphylococcus* spp.
*S*. *aureus*	*S*. *aureus*	*S*. *aureus*	*S*. *aureus*
*Streptococcus* spp.	*Streptococcus* spp.	*Streptococcus* spp.	*Streptococcus* spp.
*Streptococcus agalactiae*	EL^2^	*Streptococcus agalactiae*	*Streptococcus agalactiae*
Gram-positive^1^	*E*. *coli*	*E*. *coli*	*E*. *coli*
Gram-negative	KES^3^	Coliforms^5^	Coliforms^5^
	Gram-negative^4^	Gram-negative^6^	Gram-negative^6^

^1^ except *S*. *aureus*, *Staphylococcus* spp., *Streptococcus agalactiae* and *Streptococcus* spp.

^2^ *Enterococcus* spp. and/or *Lactococcus* spp.

^3^ *Klebsiella* spp., *Enterobacter* spp., and/or *Serratia* spp.

^4^ except *E*. *coli* and bacteria from KES group.

^5^ *Citrobacter* spp., *Klebsiella* spp., *Enterobacter* spp., and/or *Serratia* spp.

^6^ except *E*. *coli* and coliforms

### Microbiological diagnosis

Milk samples were cultured for identification of mastitis-related pathogens by the two reference laboratories as described below. In QMPS, milk samples were plated onto trypticase soy agar plates supplemented with 5% of sheep blood and 0.1% esculin using a sterile cotton swab. Plates were incubated aerobically at 35°C between 24h and 48 h. Culture characteristics evaluated included size, color, hemolytic pattern, and odor. Additional tests for further bacterial classification included Gram stain and wet mount microscopic evaluations. Biochemical tests included evaluation of the presence of catalase using 3% hydrogen peroxide, coagulase using EDTA rabbit plasma tubes, indole using SpotTest (Hardy Diagnostics), KOH string test using 3% potassium hydroxide, oxidase, lactose, sorbitol fermentation, and CAMP tests. Additionally, surface carbohydrates group typing (BactiStaph and PathoDx, Thermo Scientific) and selective differential agars such as MacConkey, Edwards, and bile esculin were used when needed. At the Veterinary Diagnostic Laboratory at University of Illinois plates were spiraled with 100 μl using a sterile “T” spreader onto TSA/5% sheep blood agar and MacConkey Agar. Then plates were incubated for 16–24 hours at 37°C in air. After 24 hours plates were read, if there are slow growing organisms plates were transferred to 37°C w/5% CO_2_. Plates were all re-incubated and re-read at 48 hours for any slow growing organisms. Plates were evaluated for quality of growth and number of organisms and ranked. The number of organisms recovered and morphologies was recorded. When the number of colony morphologies was greater than three, sample were considered contaminated unless an obvious pathogen (clear predominance of one type of colony morphology) was noticeable. Most of identifications were confirmed with matrix-assisted laser desorption/ionization-time-of-flight mass spectrometry (MALDI-TOF MS). Gram stain, catalase or oxidase Rxns were performed if needed. Traditional tubes tests TSI/LIA, Bile/Esc Salt, CAMP, coagulase was performed if an acceptable MALDI-TOF ID was not obtained. Some organisms such as mycoplasma, yeast, prototheca were identified microscopically if not found by MALDI-TOF.

Mixed infections were accounted for when two or three apparently distinct bacterial types were detected in a well-distributed growth pattern, and all pathogens were reported. Samples were considered negative if no aerobic bacterial growth was noticed within the first 48 hours of incubation following guidelines for the accredited diagnostic laboratories.

### Statistical analysis

Matched culture results between the two reference laboratories were used as Reference standard (RS) to determine the predictive factors of the on-farm culture systems. A preliminary comparative analyses was performed assigning the classification of microbiological culturing results into three larger categories (No bacterial growth, Gram-negative and Gram-positive bacteria). Further comparative analyses between plates and RS culture results were performed considering the potential microbiological diagnosing of the respective tests as indicated by the manufacturers' recommendations (Table 1). The results from all on-farm culture tests were categorized as correct or incorrect based on the RS culture results. If the diagnosis of a bacterium by a on-farm plate system was the same than RS the sample was classified as true positive (TP). If the diagnosis by an on-farm plate was no growth and RS was no growth as well the sample was classified as true negative (TN). If the diagnosis of a bacterium by on-farm plate was different than RS results (RS being different bacterium or no growth) the sample was classified as false positive (FP). If the diagnosis by on-farm plate was no growth or a different bacterium than RS specific bacterium diagnosed, the sample was classified as false negative (FN). The effectiveness for mastitis-related pathogens of each plate culture system was based on their accuracy (Ac), Sensitivity (Se), Specificity (Sp), Positive predictive value (PPV), Negative predictive value (NPV) and Cohen’s kappa coefficient (*k*). Numbers of TP, TN, FP, and FN, and number of milk samples from cows with clinical mastitis (n) were used to calculate each predictive factor as followed: Ac = (TP+TN)/n, Se = TP/(TP+FN), Sp = TN/(FP+TN), PPV = TP/(TP+FP), NPV = TN/(FN+TN). Results are expressed as total number and prevalence of pathogens, predictive factor values (Ac, Se, Sp, PPV and NPV) and 95% confidence intervals (CI). Confidence intervals were calculated based on the standard error obtained from a binomial distribution following the formulas: $\text{SE}=[\sqrt{p(1-\text{p})}]/n$ and CI = predictive factor value ± 1.96×SE, where *p* is the proportion/ prevalence of the attribute analyzed and *n* is the total number of samples analyzed. A Cohen’s kappa coefficient ≥ 0.81 corresponded to almost perfect agreement, whereas 0.61 ≤ *k* ≤ 0.80 represented a substantial agreement, an estimate ranging from 0.41 to 0.60 was considered a moderate agreement, and *k* ≤ 0.40 denoted a fair agreement.

## Results

A total of 17 and 13 different mastitis-related pathogens were identified, respectively, by the VDL and QMPS laboratories. The most common microorganisms detected by VDL laboratory were *Enterobacter* spp., *Lactococcus* spp., *Klebsiella* spp., *E*. *coli* and *Streptococcus* spp. with the prevalence of 11.9%, 8.3%, 6.7%, 6.1% and 6.1%, respectively. The QMPS laboratory predominantly isolated *Enterobacter* spp., *Streptococcus* spp., *E*. *coli* and *Klebsiella* spp. with the prevalence of 11.4%, 7.4%, 6.7% and 5.1%, respectively. Both laboratories identified a small proportion of the quarters with *Staphylococcus* spp. and *S*. *aureus* with the prevalence of ≤ 3.0% and 0.7%, respectively. No contaminated milk samples were identified by the standard laboratory analyses. For Minnesota tri-plate, SSGN, and SSGNC all false negative was derived from no growth results. For Accumast, only one of the 3 samples diagnosed as false negative had a bacterium cultured that differed from the one RS identified by RS, whereas the other two false negatives results were samples with no growth in RS.

The culture results of 211 milk samples, which had the same diagnosis between the two reference laboratories, were used as RS to calculate the predictive factors of the four plate systems (Table 2). Furthermore, the milk samples with no bacterial growth or ten distinct pathogens diagnosed by RS (*E*. *coli*, *Enterobacter* spp., *Enterococcus* spp., *Klebsiella* spp., *Lactococcus* spp., *Pseudomonas* spp., *Serratia* spp., *S*. *aureus*, *Staphylococcus* spp. and *Streptococcus* spp.) were used to calculate the predictive factors of the four plate systems.

**Table 2:** Total number (N) and prevalence (%) of distinct mastitis-associated pathogens in milk samples from cows with clinical mastitis cultured by two reference laboratories (VDL and QMPS). The total number, prevalence, and 95% confidence interval of each bacteria in the Reference Standard (RS) is presented.

	VDL^1^			QMPS^2^			RS^3^			Agreement^4^	
Bacteria	N	%	CI 95%	N	%	CI 95%	N	%	CI 95%	N/Total	%
*Enterobacter* spp.	37	11.9	8.3–15.6	34	11.4	7.8–15.0	30	14.2	9.5–18.9	30/41	73.2
*Enterococcus* spp.	3	1.0	0.0–2.0	3	1.0	0.0–2.0	3	1.4	0.1–2.1	3/3	100.0
*E*. *coli*	19	6.1	3.4–8.8	20	6.7	3.8–9.5	14	6.6	3.8–9.5	14/25	56.0
*Klebsiella* spp.	21	6.7	3.9–9.6	20	6.7	3.8–9.5	14	6.6	3.8–9.5	14/27	51.9
*Lactococcus* spp.	26	8.3	5.3–11.5	5	1.7	0.2–3.1	5	2.4	0.3–4.4	5/26	19.2
*Pseudomonas* spp.	10	3.2	1.2–5.2	7	2.4	0.6–4.0	3	1.4	0.1–3.0	3/14	21.4
*Serratia* spp.	2	0.6	0.0–1.5	2	0.7	0.2–1.6	1	0.5	0.4–1.4	1/3	33.3
*S*. *aureus*	2	0.6	0.0–1.5	2	0.7	0.2–1.6	2	0.9	0.3–2.2	2/2	100.0
*Staphylococcus* spp.	11	3.5	1.4–5.6	3	1.0	0.1–2.1	3	1.4	0.1–3.0	3/11	27.3
*Streptococcus* spp.	19	6.1	3.4–8.8	22	7.4	4.3–10.3	11	5.2	2.2–8.2	11/30	36.7
No Growth	151	48.4	43.2–54.4	174	58.6	52.6–63.8	126	59.4	53.1–66.3	126/199	63.3

^1^ Veterinary Diagnostic Laboratory (University of Illinois, Urbana, IL)

^2^ Quality Milk Production Services (Cornell University, Ithaca, NY)

^3^ Reference standard (RS) was determined by samples agreeing between the two laboratories

^4^ Agreement percent by bacteria for VDL and QMPS

The overall test characteristics and 95% confidence interval of the four culture plates for three general categories (No bacterial growth, Gram-positive and Gram-negative bacteria) and for specific mastitis-related pathogens are presented (Tables 3 and 4, respectively). Only Accumast had an almost perfect agreement (*k* = 0.81) with RS for diagnosing the three general categories. Accumast, Minnesota, SSGN and SSGNC correctly identified the mastitis-associated pathogens in 94.1%, 56.5%, 63.5% and 56.5%, respectively, of the milk samples with bacterial growth. Accumast had greater values for all predictive factors for diagnosing mastitis-related pathogens when compared to the others three plate systems (Table 4). Also, a substantial agreement (*k* = 0.79) was detected only between Accumast and RS, whereas the inter-rater agreements of Minnesota, SSGN, and SSGNC with RS were denoted moderate (0.45 ≤ *k* ≤ 0.55).

**Table 3:** Gram-positive and Gram-negative analysis with overall predictive factors and 95% confidence interval (± 95% C.I) of four plate-based culture systems (Accumast, Minnesota Easy System Tri-Plate, SSGN Quad plate and SSGNC Quad plate) according to the reference standard (agreement between the two standard laboratories).

	Plate-based culture system			
Parameter (%)	Accumast	Minnesota	SSGN	SSGNC
Accuracy	90.52±0.04	73.46±0.06	84.36±0.06	83.90±0.06
Sensitivity	97.62±0.02	88.89±0.05	78.79±0.07	82.72±0.06
Specificity	85.83±0.05	68.15±0.07	88.11±0.05	85.27±0.06
PPV^1^	82.00±0.06	48.98±0.03	75.36±0.07	77.91±0.07
NPV^2^	98.20±0.02	94.69±0.02	90.00±0.05	88.71±0.05
*k*^3^	0.81±0.01	0.53±0.01	0.66±0.01	0.67±0.01

^1^ Positive predictive value

^2^ Negative predictive value

^3^ Cohen’s kappa coefficient. κ ≤ 0 denotes poor agreement; 0.01 to 0.20 denotes slight agreement; 0.21 to 0.40 denotes fair agreement; 0.41 to 0.60 denotes moderate agreement; 0.61 to 0.80 denotes substantial agreement and 0.81 to 1.00 denotes almost perfect agreement.

**Table 4:** Bacteria associated with clinical mastitis analysis with overall predictive factors and 95% confidence interval (± 95% C.I) of four plate-based culture systems (Accumast, Minnesota Easy System Tri-Plate, SSGN Quad plate and SSGNC Quad plate) according to the reference standard (agreement between the two standard laboratories).

	Plate-based culture system			
Parameter (%)	Accumast	Minnesota	SSGN	SSGNC
Accuracy	89.57±0.04	73.46±0.06	79.15±0.07	74.88±0.07
Sensitivity	97.56±0.02	88.89±0.04	79.41±0.07	78.69±0.07
Specificity	84.50±0.05	68.15±0.07	79.02±0.07	73.33±0.07
PPV^1^	80.00±0.06	48.98±0.07	64.29±0.08	54.55±0.0
NPV^2^	98.20±0.02	94.69±0.03	88.98±0.05	89.43±0.05
*k*^3^	0.79±0.01	0.45±0.01	0.55±0.01	0.46±0.03

^1^ Positive predictive value

^2^ Negative predictive value

^3^ Cohen’s kappa coefficient. κ ≤ 0 denotes poor agreement; 0.01 to 0.20 denotes slight agreement; 0.21 to 0.40 denotes fair agreement; 0.41 to 0.60 denotes moderate agreement; 0.61 to 0.80 denotes substantial agreement and 0.81 to 1.00 denotes almost perfect agreement.

The predictive factors for species of bacteria or group of bacteria identified by each plate system that were found in at least 10 samples are described in Tables 5–8. Bacteria that had low prevalence in the study samples are not presented to prevent the possibility of making inaccurate inferences. The use of all plate tests for bacteria present in at least 10 samples resulted in higher Ac for Accumast (93.8% to 96.8) than Minnesota Tri-plate (80.2% to 89.8%), SSGN (83.6% to 93.1%), and SSGNC (85.3% to 90.3%). Likewise, Accumast had high Se (87.5% to 100%) and Sp (93.2% to 96.5%) than Minnesota Tri-plate Se (55.6% to 72.7%) and Sp (82.9% to 93.9%), SSGN Se (0% to 83.3%) and Sp (83.7% to 94.2%), and SSGNC Se (0% to 73.3%) and Sp (87.3% to 95.7%). The four on-farm culture systems showed high Ac for bacteria belonging to the *Streptococcus* spp. (Ac ≥ 89.3%). But, Accumast resulted in a Se of 100% for *Streptococcus* spp., whereas Minnesota tri-plate, SSGN, and SSGNC had Se of 72.7%, 63.6% and 63.6%, respectively. The Ac and Sp of Accumast, SSGN, and SSGNC to *E*. *coli* mastitis were high (Ac ≥ 88.9% and Sp ≥ 92.4%).

**Table 5:** Prevalence and predictive factors (± 95% C.I) of Accumast for bacteria associated with clinical mastitis found in at least 10 samples according to the reference standard (agreement between the two standard laboratories).

		Bacteria or group of bacteria identified by Accumast			
Parameter	*E*. *coli*	KES^1^	EL^2^	*Streptococcus* spp.	No Growth
Number	18	49	13	20	111
Prevalence (%)	8.2 (4.6–11.8)	22.4 (16.8–27.8)	5.9 (2.8–9.0)	9.1 (5.3–12.9)	50.5 (43.8–57.1)
Accuracy (%)	96.8±0.04	97.5±0.04	94.3±0.12	93.8±0.10	85.2±0.05
Sensitivity (%)	100±0.14	97.8±0.04	87.5±0.17	100±0.13	0.0±0.00
Specificity (%)	96.5±0.08	96.5±0.05	94.8±0.12	93.2±0.11	86.5±0.04
PPV^3^ (%)	76.5±0.19	91.8±0.07	53.8±0.26	60.0±0.20	0.0±0.00
NPV^4^ (%)	100±0.14	99.1±0.02	99.1±0.05	100±0.13	98.2±0.02

^1^ *Klebsiella* spp., *Enterobacter* spp. or *Serratia* spp.

^2^ *Enterococcus* spp. or *Lactococcus* spp.

^3^ Positive predictive value

^4^ Negative predictive value

**Table 6:** Prevalence and predictive factors (± 95% C.I) for Minnesota Easy System Tri-plate associated with clinical mastitis found in least 10 samples according to the reference standard (agreement between the two standard laboratories).

	Bacteria or group of bacteria identified by Minnesota Easy System			
Parameter	Gram-negative	Gram-positive^1^	*Streptococcus* spp.	No Growth
Number	42	28	18	113
Prevalence (%)	19.7 (14.4–25.1)	13.1 (8.6–17.7)	8.5 (4.7–12.2)	53.1 (46.3–59.7)
Accuracy (%)	80.2±0.11	81.3±0.13	89.8±0.13	81.1±0.05
Sensitivity (%)	55.6±0.13	60.0±0.17	72.7±0.20	0.0±0.00
Specificity (%)	93.9±0.06	82.9±0.13	91.5±0.12	84.9±0.05
PPV^2^ (%)	83.3±0.17	21.4±0.14	44.4±0.22	0.0±0.00
NPV^3^ (%)	79.3±0.13	96.4±0.06	97.3±0.07	94.7±0.03

^1^ Except *Staphylococcus aureus*, *Staphylococcus* spp., *Streptococcus agalactiae* and *Streptococcus* spp.

^2^ Positive predictive value

^3^ Negative predictive value

**Table 7:** Prevalence and predictive factors with 95% confidence interval (± 95% C.I) of SSGN Quad plate to identify bacteria associated with clinical mastitis found in least 10 samples according to the reference standard (agreement between the two standard laboratories).

	Bacteria or group of bacteria identified by SSGN Quad plate			
Parameter	Coliforms^1^	*Staphylococcus* spp.	*Streptococcus* spp.	No Growth
Number	52	14	14	127
Prevalence (%)	24.3 (18.5–30.0)	6.5 (3.2–9.9)	6.5 (3.2–9.9)	59.3 (52.7–65.9)
Accuracy (%)	83.6±0.08	87.0±0.17	93.1±0.13	80.7±0.04
Sensitivity (%)	83.3±0.08	0.0±0.00	63.6±0.24	0.0±0.00
Specificity (%)	83.7±0.08	89.0±0.16	94.2±0.12	89.7±0.03
PPV^2^ (%)	61.4±0.11	0.0±0.00	50.0±0.25	0.0±0.00
NPV^3^ (%)	94.2±0.05	96.6±0.09	96.6±0.09	89.0±0.03

^1^ *Citrobacter* spp., *Enterobacter* spp., *Klebsiella* spp. and/or *Serratia* spp.

^2^ Positive predictive value

^3^ Negative predictive value

**Table 8:** Prevalence and predictive factors with 95% confidence interval (± 95% C.I) of SSGNC Quad plate to identify bacteria associated with clinical mastitis found in least 10 samples according to the reference standard (agreement between the two standard laboratories).

	Bacteria or group of bacteria identified by SSGNC Quad plate				
Parameter	*E*. *coli*	Coliforms^1^	*Staphylococcus* spp.	*Streptococcus* spp.	No Growth
Number	21	35	16	17	123
Prevalence (%)	9.7 (5.7–13.7)	16.2 (11.3–21.1)	7.4 (3.9–10.9)	7.9 (4.3–11.5)	56.9 (50.3–63.5)
Accuracy (%)	90.3±0.12	87.5±0.10	85.3±0.17	89.3±0.14	79.1±0.05
Sensitivity (%)	73.3±0.18	64.4±0.15	0.0±0.00	63.6±0.22	0.0±0.00
Specificity (%)	92.4±0.11	95.7±0.06	87.3±0.16	91.7±0.13	87.3±0.04
PPV^2^ (%)	55.0±0.21	85.3±0.11	0.0±0.00	41.2±0.22	0.0±0.00
NPV^3^ (%)	96.5±0.08	87.3±0.10	97.3±0.08	96.5±0.09	89.4±0.04

^1^ *Citrobacter* spp., *Enterobacter* spp., *Klebsiella* spp. and/or *Serratia* spp.

^2^ Positive predictive value

^3^ Negative predictive value

## Discussion

This study was the first that compared four commercial on-farm culture systems’ effectiveness for the identification of specific pathogens associated with clinical mastitis in dairy cattle. All parameters used as criteria to determine the accuracy of tests used showed a higher agreement between the Accumast system with the standard laboratory analyses used as reference standard than the other on-farm methods utilized. Our findings suggest that although all the on-farm culture systems are capable of identifying mastitis pathogens, their accuracy varies and farm personnel and veterinarians should consider this information when making decisions on therapeutic selection or management of clinical cases on a dairy farm.

As expected, most of misclassification that lead to false positive results were derived from no growth samples in the RS that had culture positive for the on-farm culture plates. Most of the false negative results were no growth samples for on-farm plates that grew bacteria in RS. The most common causes of false positive were Lactococcus/Enterococcus for Accumast, Gram-positive and Gram-negative for Minnesota tri-plate, and *Staphylococcus* spp. for SSGN and SSGNC. It is noteworthy that the number of false positives (n = 19) for Accumast was remarkably lower than for Minnesota tri-plate (n = 45), SSGN (n = 27) and SSGNC (n = 36), respectively. The most common cause of false negative for Minnesota tri-plate, SSGN, and SSGNC were *Enterobacter*, *Lactococcus*, *Klebsiella*, *Pseudomonas*, a group of four bacteria that Accumast was able to identify in all except 2 samples. The increased ability of Accumast to identify *Enterobacter* spp., *Lactococcus* spp., *Klebsiella* spp., *Pseudomonas* spp. compounded to its lower number of false positives explains in part its higher accuracy, specificity, sensitivity, PPV, NPV, and kappa-values.

Previous studies have utilized a single NMC laboratory culturing result as the RS to analyze the accuracy of on-farm culture systems [19,21]. Also, the bacterial 16S rRNA sequencing is an alternative to characterize the microbial profile of milk samples derived from healthy, subclinically and clinically diseased cows [25]. However, the use of this method as a RS for on-farm culture analyzes does not permit the calculation of specific predictive values because there are no negative test results [20]. To optimize the presentation of the data in the manuscript, we opted to present the results that agreed between the two laboratories as RS. It is noteworthy that the laboratories used different protocols for the microbiological analyses. The discrepancies found between the laboratories might be a result of using different protocols. Future studies should investigate further discrepancies amongst laboratories to reduce inconsistency of methodologies and diagnosis. Moreover, a contributor for the differences between laboratories and on-farm culture is the nature of the milk samples used in this study. Samples used for microbiological analyzes in the laboratories were frozen, a common fact in routine samples received in milk quality laboratories, whereas sample used from on-farm samples were kept on ice before culture. It is reasonable to surmise that this scenario represents the reality of most dairy industry and further research needs to address how it contributes to the discrepancies of results between samples analyzed in milk quality laboratories and farms.

Most of the on-farm tests yielded negative cultures or coliform growth, as expected [3, 4, 26]. Despite a potential presence of inhibitory substances from the inflammation or a low amount of bacteria that may elude the microbiological diagnosis, the identification of no growth cultures have been previously attributed to the ability of the immune system to promptly eradicate the primary pathogen without the need of intramammary antibiotics [27]. Also, the clinical mastitis signs may be seldom caused by organisms not grown by routine microbiological tests, such as *Mycoplasma* spp. [28, 29]. Our results indicate the effectiveness of the four on-farm systems for the early identification of clinical mastitis associated with no growth cultures, which is a critical step to reduce antibiotic usage in dairy herds [30, 31].

Several studies have reported the spontaneous self-cure of mild to moderate *E*. *coli* mastitis cases [13, 32, 33]. On the other hand, *Klebsiella* intrammamary infections have been associated with a more severe innative immune response [34, 35], a prolonged milk production loss [26, 36], a larger risk of culling [37] and a reduced rate of spontaneous self-cure [14] than the other coliforms mastitis cases. Despite its severity, the proper treatment of *Klebsiella* intramammary infections may result in an important increase in the bacterial cure [14, 38, 39]. Furthermore, the prompt diagnosis of *Klebsiella* mastitis cases may be imperative for implementation of preventive approaches, targeting the decrease of exposure and to limit the risk of udder infections [40]. In this regard, the four on-farm systems presented a similarly high ability to identify culture-negative bovine clinical mastitis samples. However, only the Accumast system had a sensitivity equal or above 96.8% to detect *E*. *coli*, *Pseudomonas* spp. and KES mastitis. The modest ability to correctly identify specific Gram-negative colonies presented by Minnesota and both Quad plate systems may affect the rational use of antibiotics, resulting in needless or privation of treatments of affected cows [27].

Antimicrobial therapies are often applied to cows experiencing clinical mastitis caused by Gram-positive pathogens [14]. However, the prompt identification of *Staphylococcus aureus* infections is essential for the management of dairy herds due to their poor response to intramammary antimicrobial treatments [41–44]. Unfortunately, only 3 samples were positive for *S*. *aureus* in RS samples in the study, making the finding related to this bacterium difficult to assess. The diagnosis of the two positive cases presented by Accumast concurred with previous studies using chromogenic methods for isolation and identification of *Staphylococcus aureus* [20, 45]. The absence of *S*. *aureus* positive diagnosis by Minnesota, SSGN, and SSGNC may be attributed to their media composition. Blood agar systems indicate *S*. *aureus* by the presence of β-hemolysis [46]. However, many isolates of this species do not generate detectable hemolysis zones in primary cultures [47]. Besides the inability to identify milk samples infected with *S*. *aureus*, Minnesota and both Quad-plates also showed a high number of incorrect positive diagnosis. Streptococci and non-*aureus* species of staphylococci colonies can induce media phenomenon similar to β-hemolysis in blood agar systems, leading to uncertain interpretation [19]. Considering its relatively low probability of cure [43, 44], an increased number of false positive diagnosis of *S*. *aureus* cases may lead to the unnecessary segregation and the premature culling of cows [48], which represent major economic costs of clinical mastitis. *Staphylococcus* spp. are a major cause of persistent intramammary infection [49], impaired udder health, and reduced milk production [50]. Considering the different responsiveness to antibiotic therapies of *Staphylococcus* spp. when compared with *S*. *aureus* [15], the proper identification of *Staphylococcus* spp. infections can be pivotal to reduce a selective pressure and the dissemination of resistant pathogens [51]. Unfortunately, the limited ability to identify infections with non-aureus species of staphylococci observed in the present study is a limitation of the four on-farm culture systems.

A prompt diagnosis followed by the correct intramammary therapy is needed for the efficient management of clinical mastitis caused by environmental *streptococci* [27, 52]. In spite of the potential resistance to specific antimicrobials [53], *Streptococcus* spp. infections are generally sensitive to multiple formulations, resulting in a high rate of cure in a cost effective manner [11]. Moreover, the markedly antimicrobial susceptibility variance between isolates of *Staphylococcus aureus* and *Streptococcus* spp. [12, 54] encourages the diagnosis at more specific levels of Gram-positive infections. Surprising, Minnesota Tri-Plate was not effective to differentiate *Streptococcus* spp. from other organisms as previously reported [55]. However, our results confirmed the high accuracy of Accumast for identification of environmental streptococci independent of the species [20].

Our findings suggest that Accumast was efficient identifying mastitis-related pathogens, as previously indicated [20]. However, this attribute of Accumast must be carefully interpreted, because some pathogens such as *Staphylococcus aureus* were present in a very small number making any extrapolations difficult. Derived blood agar plates conventionally allow the growth of an extensive range of bacteria, but rarely permit more than a presumptive diagnosis according to colony appearance [56]. Conversely, the chromogenic media can identify pathogens with high accuracy by the inclusion of chromogenic enzyme substrates targeting specific microbial proteins [57, 58]. Nonetheless, the preventive and therapeutic management of many dairy farms is still based on the diagnosis at general levels (Gram-negative, Gram-positive and No Growth). When analyses were categorized at the general level, Accumast remained as the test with highest sensitivity, positive predictive value, negative predictive value, accuracy and the only test with almost perfect agreement as measured by Cohen-K value. However, specificity, the ability of finding true negative cases, was the highest for SSNG, with SSGNC and Accumast having similar outcomes. These results suggest that in farms where mastitis treatment decision remains based on Gram-positive and Gram-negative diagnosis, the ability to find negative cows will not benefit from using Accumast.

## Conclusion

Under the current conditions, Accumast was the most accurate plate test for diagnosing of mastitis-related pathogens, showing greater values for all overall predictive factors and a substantial agreement with the laboratory culture result. Minnesota Easy System Tri-plate, SSGN and SSGNC Quad plates had inconsistent results when classification at the genus and species level was attempted. On the other hand, the chromogenic media of Accumast were a powerful tool to diagnosis specific microorganisms, such as *E*. *coli* and bacteria belonging to *Streptococcus* spp. Nevertheless, all plate systems were highly efficient to identify milk samples with no microbiological growth. When diagnosis is based on Gram-positive and Gram-negative, Accumast remained the best test for all parameters with exception of specificity.
